# Cross-population heterogeneity in the association between estimated sdLDL-C and gallstone disease: a dual-source two-sample cross-sectional study

**DOI:** 10.3389/fpubh.2026.1922892

**Published:** 2026-09-07

**Authors:** Wei Dai, Weihao Guo, Hang Shen, Qikai Sun, Yanqun Duan, Yeben Qian

**Affiliations:** 1 Department of Hepatobiliary and Pancreatic Surgery, The First Affiliated Hospital of Anhui Medical University, Hefei, Anhui, China; 2 Department of Dermatology, The First Affiliated Hospital of Anhui Medical University, Hefei, Anhui, China

**Keywords:** cross-population analysis, estimated small dense LDL cholesterol, gallstone disease, lipid metabolism, threshold effect

## Abstract

**Background:**

While conventional lipid parameters are linked to gallstone disease, the specific role of small dense low-density lipoprotein cholesterol (sdLDL-C)—a highly atherogenic subfraction—remains unexplored. We aimed to evaluate the independent association between estimated sdLDL-C and gallstone risk, and to map potential cross-population dose-response relationships.

**Methods:**

This dual-source cross-sectional study integrated a strictly defined Chinese clinical sample (*n* = 730, comprising 334 symptomatic surgical cases and 396 healthy controls from routine health check-ups) and the US NHANES database (*n* = 6,799). To facilitate large-scale, cost-effective screening, serum sdLDL-C was estimated using the internationally validated Sampson equation, strictly excluding subjects with extreme hypertriglyceridemia (TG ≥ 800 mg/dL). Multivariate logistic regression and two-piecewise models were utilized to evaluate independent associations and potential non-linear patterns. Sensitivity analyses rigorously excluded patients with major metabolic comorbidities (hypertension, diabetes, and clinical dyslipidemia).

**Results:**

In the multivariable-adjusted models, when analyzed as a continuous variable, elevated estimated sdLDL-C was significantly and positively associated with gallstone risk in both the Chinese sample (OR = 1.12, 95% CI: 1.08–1.15) and the NHANES cohort (OR = 1.03, 95% CI: 1.01–1.04). Sensitivity analysis excluding individuals with diabetes and hypertension confirmed the stability of this positive association (OR = 1.11, 95% CI: 1.07–1.15). Dose-response analysis revealed a non-linear threshold effect in the Chinese sample, with an inflection point at 34.51 mg/dL (sdLDL-C ≥ 34.51 mg/dL: OR = 1.27, 95% CI: 1.20–1.34, *p* < 0.001), whereas a steady linear association was observed in the NHANES population. Stratified analyses indicated that this positive association was particularly prominent in females and diabetic individuals.

**Conclusion:**

Estimated sdLDL-C exhibits an independent and positive association with gallstone disease, suggesting it might be more than merely a secondary manifestation of late-stage metabolic syndrome. The identification of a specific non-linear threshold in the Chinese population, juxtaposed with a linear pattern in the US, suggests the potential value of exploring sdLDL-C for risk stratification in biliary diseases, though these cross-sectional findings require prospective validation.

## Introduction

Gallstones are among the most common gastrointestinal and biliary disorders worldwide. Their pathogenesis is multifactorial, involving genetic, environmental, lifestyle, and metabolic factors (1). With shifts in modern dietary patterns and rising obesity rates, the risk of gallstones continues to increase. This not only impairs patients’ quality of life but also imposes a substantial economic burden on global healthcare systems. Extensive epidemiological studies have shown that gallstone disease is highly comorbid with metabolic syndrome and hepatic metabolic dysfunction, such as non-alcoholic fatty liver disease (2, 3). Therefore, identifying novel metabolic biomarkers for potential risk prediction is of great clinical importance for screening and intervening in high-risk populations.

Lipid metabolism disorders, particularly the loss of cholesterol homeostasis, are widely recognized as the core pathological mechanism of cholesterol gallstones. Recent multicenter studies have confirmed significant associations between various abnormal lipid parameters and gallstone risk (4, 5). Traditional lipid metrics, such as total cholesterol (TC) and low-density lipoprotein cholesterol (LDL-C), are commonly used in clinical risk assessment. However, LDL particles are highly heterogeneous in size, density, and atherogenic potential, meaning that the total LDL-C level often fails to fully capture the depth of an individual’s lipid dysregulation. In recent years, small dense low-density lipoprotein cholesterol (sdLDL-C), a specific subfraction of LDL-C, has drawn attention. Small dense LDL particles are typically defined by a greater density and a smaller diameter compared to large buoyant LDL. The Sampson equation utilized in this study mathematically captures this specific subfraction by modeling the distribution of triglycerides and cholesterol among lipoprotein particles (6). Because it is smaller, denser, and highly susceptible to oxidation, sdLDL-C is considered more atherogenic than standard LDL-C and a better indicator of underlying metabolic abnormalities (7).

Although the predictive value of sdLDL-C in cardiovascular diseases and metabolic syndrome is well established, its specific role and clinical significance in gallstone pathogenesis remain to be systematically elucidated, and its dose-response relationship with gallstone risk across distinct populations remains to be fully mapped. While previous epidemiological studies and meta-analyses have reported associations between conventional lipid markers, atherogenic indices, and gallstone disease (3, 4), the specific dose-response characteristics of sdLDL-C are less clear.

Given that cholesterol gallstone formation is fundamentally linked to abnormal biliary lipid secretion and systemic lipid imbalance, we hypothesized that elevated estimated serum sdLDL-C levels are independently and positively associated with the risk of prevalent gallstone disease. To test this hypothesis, we utilized a dual-center dual-source two-sample cross-sectional design integrating clinical data from a Chinese single-center cohort sample and a large population sample from the US NHANES. By utilizing the validated Sampson equation, we aimed to systematically evaluate these associations and, crucially, explore potential population-specific non-linear dose-response relationships and thresholds. Furthermore, we employed the internationally validated Sampson equation to estimate sdLDL-C levels in large cohorts (6) and explored the potential non-linear dose-response relationships and threshold effects, providing new evidence for lipid management in gallstone disease.

## Materials and methods

### Study population and ethical statement

This study utilized a dual-source retrospective two-sample cross-sectional design. First, clinical data were collected consecutively from the First Affiliated Hospital of Anhui Medical University, constituting a hospital-based case–control study. The case group consisted of patients who underwent surgical treatment for gallstone disease at the Department of Hepatobiliary and Pancreatic Surgery between March 2022 and September 2025. The control group consisted of contemporaneous healthy individuals undergoing routine physical examinations at the Health Management Center of the same hospital (defined as individuals without evidence of gallstones on ultrasound and free from severe systemic diseases based on medical history).

**Figure 1:**
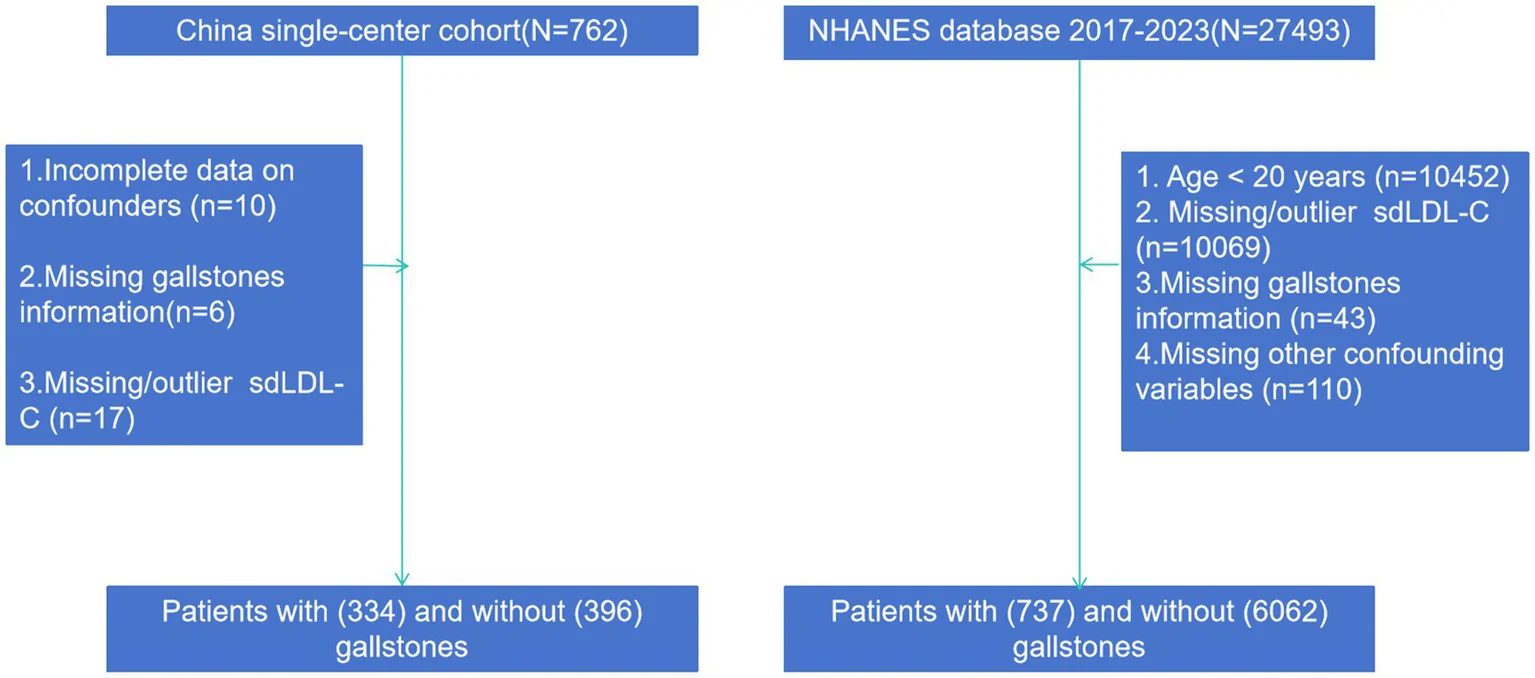
Flowchart of participant selection.

Following strict pre-specified inclusion and exclusion criteria, 730 subjects were included, comprising 334 symptomatic surgical gallstone cases and 396 healthy controls. To ensure the validity of the control group, all 396 control subjects were required to have systematic negative hepatobiliary ultrasound imaging within 1 month of blood sampling, effectively ruling out asymptomatic undiagnosed gallstones. Similarly, all cases had their gallstone diagnosis confirmed by positive hepatobiliary imaging within 1 month prior to surgery and blood sampling, ensuring temporal comparability. The exclusion criteria for both groups were: (1) severe liver disease (e.g., cirrhosis, viral hepatitis); (2) known malignancies; (3) current pregnancy; (4) acute inflammatory illness; and (5) current use of lipid-altering medications (such as statins, fibrates, ezetimibe) or medications known to affect gallstone risk. This part of the study was approved by the Ethics Committee of the First Affiliated Hospital of Anhui Medical University, and written informed consent was obtained from all participants (Figure 1).

To validate the generalizability and stability of the findings across populations, we also included data from the NHANES database. NHANES is an ongoing survey designed to assess the health and nutritional status of adults and children in the United States, and its protocols were approved by the National Center for Health Statistics (NCHS) Research Ethics Review Board. After matching (excluding participants with missing lipid data for sdLDL-C calculation, unknown gallstone status, or incomplete covariate data), the NHANES cohort included 6,799 participants, consisting of 737 gallstone patients and 6,062 non-stone participants (Figure 1).

### Data collection and variable definitions

For the Chinese cohort, demographic data (age, sex) and medical history (hypertension, diabetes) were extracted from the electronic medical record system by trained researchers blinded to the study’s lipid hypothesis. Fasting venous blood samples were collected after an overnight fast of at least 8 h. Gallstone diagnosis for cases was based on preoperative hepatobiliary ultrasound or computed tomography (CT) as the gold standard, and all included cases were symptomatic patients who subsequently underwent cholecystectomy or gallstone surgery. This strictly defined phenotype ensures that the case group represents clinically significant, symptomatic gallstone disease.

For the NHANES cohort, all laboratory data were measured by standardized laboratories of the Centers for Disease Control and Prevention (CDC), and the data collection process included covariates consistent with the Chinese cohort. The diagnosis of gallstones in NHANES was based on the Medical Conditions Questionnaire (MCQ), specifically an affirmative answer to the question, “Has a doctor or other health professional ever told you that you had gallstones?” This self-reported standard has been widely validated for its reliability in large epidemiological studies.

### Assessment of sdLDL-C

Because direct measurement of sdLDL-C is costly and difficult to implement in large retrospective cohorts, this study used the widely accepted and rigorously validated Sampson equation to calculate serum sdLDL-C levels (6). The specific formula applied was: ElbLDLC = 1.43 × LDLC − (0.14 × (ln(TG) × LDLC)) − 8.99, EsdLDLC = LDLC − ElbLDLC. All baseline lipid parameters, including directly measured LDL-C, TG, and TC, were obtained from fasting venous blood samples and expressed in mg/dL. This equation has been validated for population estimation and shows high concordance with the gold-standard ultracentrifugation method, specifically for individuals with fasting TG levels < 800 mg/dL (6). Accordingly, any subjects in both the Chinese (*n* = 1 excluded) and NHANES (*n* = 0 excluded) samples with extreme hypertriglyceridemia (TG ≥ 800 mg/dL) falling outside this valid analytical range were excluded prior to statistical modeling.

### Statistical analysis

Continuous variables were expressed as mean ± standard deviation (SD) based on their distribution (assessed using the Shapiro–Wilk test), and group comparisons were performed using the independent Student’s *t*-test (with homogeneity of variances verified by Levene’s test; Welch’s *t*-test was applied when variances were unequal). Categorical variables were presented as frequencies and percentages (%), and group comparisons were conducted using the Chi-square test.

To appropriately account for the complex multistage probability sampling design of NHANES, all analytical procedures for the US database strictly incorporated primary sampling units (PSU), strata, and survey weights to produce nationally representative estimates and standard errors. Specifically, because the estimation of sdLDL-C requires fasting triglycerides, the specific fasting subsample weights were applied rather than the full-sample weights, in accordance with NHANES analytical guidelines. Taylor series linearization was used for variance estimation. To evaluate the independent association between estimated sdLDL-C and gallstone risk, we constructed sequential multiple logistic regression models. To prevent severe mathematical coupling, the direct operational lipid components utilized in the Sampson estimation framework (specifically LDL-C, TG, and HDL-C) were explicitly excluded from the covariate adjustment sets, including all sensitivity analyses. Conversely, Total Cholesterol (TC) was retained in the fully adjusted model. Clinically, adjusting for TC controls for the baseline effect of overall hypercholesterolemia, thereby allowing us to isolate the specific atherogenic contribution of the sdLDL-C subfraction (lipid quality) from the total cholesterol burden (lipid quantity). Statistically, collinearity diagnostics confirmed that retaining TC did not induce severe multicollinearity, as the Variance Inflation Factors (VIF) for all included covariates in Model 3 were strictly less than 5. To evaluate the independent association between sdLDL-C and gallstone risk, we constructed sequential multivariate logistic regression models:

Model 1: Unadjusted crude model.

Model 2: Adjusted for core demographic variables (age and sex).

Model 3: Further adjusted for all relevant baseline covariates (e.g., BMI, ALT, albumin, total cholesterol, SII, and comorbidities like hypertension and diabetes) selected based on univariate associations and prior literature. Model 4 (Sensitivity Analysis): To eliminate the confounding effects of severe systemic lipid metabolism disorders, this model specifically excluded patients diagnosed with hypertension and diabetes across both cohorts. Other metabolic conditions, such as continuous BMI (obesity), were adjusted for in the models rather than excluded to maintain sufficient statistical power.

Subgroup analyses stratified by age (20–55 vs. 55–85 years), sex, hypertension, and diabetes were performed to test for potential heterogeneity. Finally, to explore whether a non-linear dose-response relationship existed, we applied smooth curve fitting using restricted cubic splines (RCS). A two-piecewise regression model and a log-likelihood ratio test were then used to accurately calculate the risk inflection point (threshold effect).

All statistical analyses were performed using R software (version 4.2.2) and EmpowerStats software. A two-sided *p*-value < 0.05 was considered statistically significant.

## Results

### Baseline characteristics of the Chinese cohort

The Chinese single-center cohort included 730 subjects: 334 gallstone patients and 396 controls. As shown in Table 1, there were no significant differences between the two groups in age, albumin, AST, sex distribution, or the prevalence of hypertension and diabetes (all *p* > 0.05). However, the gallstone group had a significantly lower BMI compared to the control group (25.51 ± 3.71 vs. 24.66 ± 3.44 kg/m^2^, *p* = 0.008). Regarding metabolic parameters, the gallstone group had significantly higher ALT levels (27.78 ± 24.86 U/L vs. 23.38 ± 18.09 U/L; *p* = 0.006) and total cholesterol levels (182.18 ± 37.84 mg/dL vs. 164.37 ± 29.70 mg/dL; *p* < 0.001) than the control group. Notably, serum sdLDL-C levels were also significantly higher in gallstone patients (38.72 ± 11.51 mg/dL vs. 31.21 ± 7.22 mg/dL; *p* < 0.001).

**Table 1:** Baseline data for populations in local regions of China.

Characteristic	Non-stone formers	Stone formers	*p*-value
*N*	396	334	
Age (years)	50.78 ± 13.25	50.74 ± 11.25	0.967
BMI (kg/m^2^)	25.51 ± 3.71	24.66 ± 3.44	0.008
Albumin (g/L)	43.64 ± 4.05	44.01 ± 4.46	0.234
ALT (U/L)	23.38 ± 18.09	27.78 ± 24.86	0.006
AST (U/L)	21.19 ± 12.01	22.42 ± 12.88	0.184
Serum cholesterol (mg/dl)	164.37 ± 29.70	182.18 ± 37.84	<0.001
SII	545.92 ± 542.10	537.65 ± 706.58	0.858
sdLDL-C	31.21 ± 7.22	38.72 ± 11.51	<0.001
Gender (%)			0.511
Male	230 (58.08%)	202 (60.48%)	
Female	166 (41.92%)	132 (39.52%)	
High blood pressure (%)			0.751
No	277 (69.95%)	230 (68.86%)	
Yes	119 (30.05%)	104 (31.14%)	
Diabetes (%)			0.911
No	332 (83.84%)	279 (83.53%)	
Yes	64 (16.16%)	55 (16.47%)	

### Baseline characteristics of the NHANES cohort

To validate these findings across populations, 6,799 subjects from the NHANES database were included, 737 of whom reported a history of gallstones. As shown in Table 2, compared to the non-stone group, gallstone patients in the NHANES cohort were older (59.20 ± 15.24 vs. 51.57 ± 17.32 years) and had a higher BMI (33.29 ± 8.61 vs. 29.44 ± 7.22 kg/m2) (both *p* < 0.001). Laboratory tests showed lower albumin levels and a higher SII in the gallstone group. Consistent with the Chinese cohort, sdLDL-C levels were significantly elevated in the gallstone group (33.23 ± 13.35 mg/dL vs. 31.85 ± 12.22 mg/dL; *p* = 0.004). Moreover, the proportions of females (72.86% vs. 51.07%), hypertension (51.97% vs. 36.72%), and diabetes (24.42% vs. 13.76%) were significantly higher among gallstone patients (all *p* < 0.001).

**Table 2:** Baseline population data from the US NHANES database.

Characteristic	Non-stone formers	Stone formers	*p*-value
*N*	6,062	737	
Age (years)	51.57 ± 17.32	59.20 ± 15.24	<0.001
BMI (kg/m^2^)	29.44 ± 7.22	33.29 ± 8.61	<0.001
Serum cholesterol (mg/dl)	185.13 ± 40.82	183.77 ± 43.88	0.396
SII	538.26 ± 340.83	566.79 ± 328.23	0.031
ALT (U/L)	21.87 ± 18.18	21.80 ± 16.42	0.917
Albumin (g/L)	40.36 ± 3.38	39.10 ± 3.41	<0.001
AST (U/L)	22.01 ± 14.01	21.91 ± 13.38	0.865
sdLDL-C	31.85 ± 12.22	33.23 ± 13.35	0.004
Gender (%)			<0.001
Male	2,966 (48.93%)	200 (27.14%)	
Female	3,096 (51.07%)	537 (72.86%)	
High blood pressure (%)			<0.001
Yes	2,226 (36.72%)	383 (51.97%)	
No	3,836 (63.28%)	354 (48.03%)	
Diabetes (%)			<0.001
Yes	834 (13.76%)	180 (24.42%)	
No	5,228 (86.24%)	557 (75.58%)	

All statistical estimates (including means, frequencies, and odds ratios) for the NHANES cohort are survey-weighted to represent the US national population.

### Association between sdLDL-C and gallstone risk, and sensitivity analysis

Sequential multiple logistic regression models were employed to rigorously evaluate the independent association between estimated sdLDL-C and gallstone risk (Table 3). When modeled as a continuous variable, the fully adjusted model (Model 3) revealed a statistically significant positive association across both datasets. Specifically, each 1 mg/dL increment in sdLDL-C corresponded to a 3% elevated risk of gallstones in the NHANES cohort (OR = 1.03, 95% CI: 1.01–1.04) and a 12% increased risk in the Chinese hospital-based sample (OR = 1.12, 95% CI: 1.08–1.15).

**Table 3:** Association between sdLDL-C and gallstone risk: Multivariable logistic regression analysis in Chinese and NHANES populations.

Characteristic	Model 1 OR (95% CI)	Model 2 OR (95% CI)	Model 3 OR (95% CI)	Model 4 OR (95% CI)
China data				
sdLDL-C	1.09 (1.07, 1.11)	1.09 (1.07, 1.11)	1.12 (1.08, 1.15)	1.11 (1.07, 1.15)
Categories				
Low	1	1	1	1
Middle	0.86 (0.58, 1.26)	0.85 (0.58, 1.25)	0.85 (0.55, 1.31)	0.87 (0.51, 1.49)
High	6.78 (4.55, 10.11)	6.82 (4.57, 10.18)	6.69 (3.80, 11.78)	4.90 (2.49, 9.62)
NHANES data				
sdLDL-C	1.01 (1.00, 1.01)	1.01 (1.00, 1.01)	1.03 (1.01, 1.04)	1.03 (1.01, 1.04)
Categories				
Low	1	1	1	1
Middle	1.26 (1.04, 1.52)	1.15 (0.95, 1.39)	1.14 (0.92, 1.41)	1.11 (0.79, 1.56)
High	1.19 (0.98, 1.44)	1.11 (0.91, 1.35)	1.19 (0.90, 1.59)	1.24 (0.81, 1.90)

Model 1 = No covariates were adjusted.

Model 2 = Model 1 + age, gender.

Model 3 was adjusted for all covariates in Tables 1 or 2.

Model 4 was adjusted for the same variables as Model 3 and excluded individuals with hypertension and diabetes.

All statistical estimates (including means, frequencies, and odds ratios) for the NHANES cohort are survey-weighted to represent the US national population.

Categorical variables were defined based on tertiles of sdLDL-C (mg/dL). Due to the exclusion of specific populations in Model 4, the tertile cut-offs differ slightly from Models 1–3. For China Data: Models 1–3 cut-offs: low (12.56–29.96), Middle (29.96–38.16), high (38.16–91.11). Model 4 cut-offs: low (12.56–29.12), middle (29.12–37.43), high (37.43–73.84). For NHANES Data: Models 1–3 cut-offs: low (3.18–25.42), middle (25.42–35.47), high (35.47–122.48). Model 4 cut-offs: low (3.18–25.16), middle (25.16–35.68), high (35.68–101.98).

To enforce strict methodological control, we performed a sensitivity analysis (Model 4) by explicitly excluding subjects diagnosed with severe metabolic comorbidities, namely hypertension and diabetes. Despite these stringent sample restrictions, the independent positive association remained highly stable in the Chinese dataset (OR = 1.11, 95% CI: 1.07–1.15). Furthermore, to cross-verify the effect consistency, sdLDL-C was analyzed categorically based on tertiles. While the highest tertile consistently indicated an elevated risk, the categorically analyzed Model 4 in the Chinese cohort yielded an OR of 4.90 with a conspicuously wide confidence interval (95% CI: 2.49–9.62). This wide interval reflects statistical instability secondary to sparse-data bias within specific strata following the strict exclusions. Consequently, the continuous variable analysis is deemed the more statistically rigorous and reliable approach for effect estimation in this study.

### Dose-response relationship and threshold effect analysis

**Figure 2:**
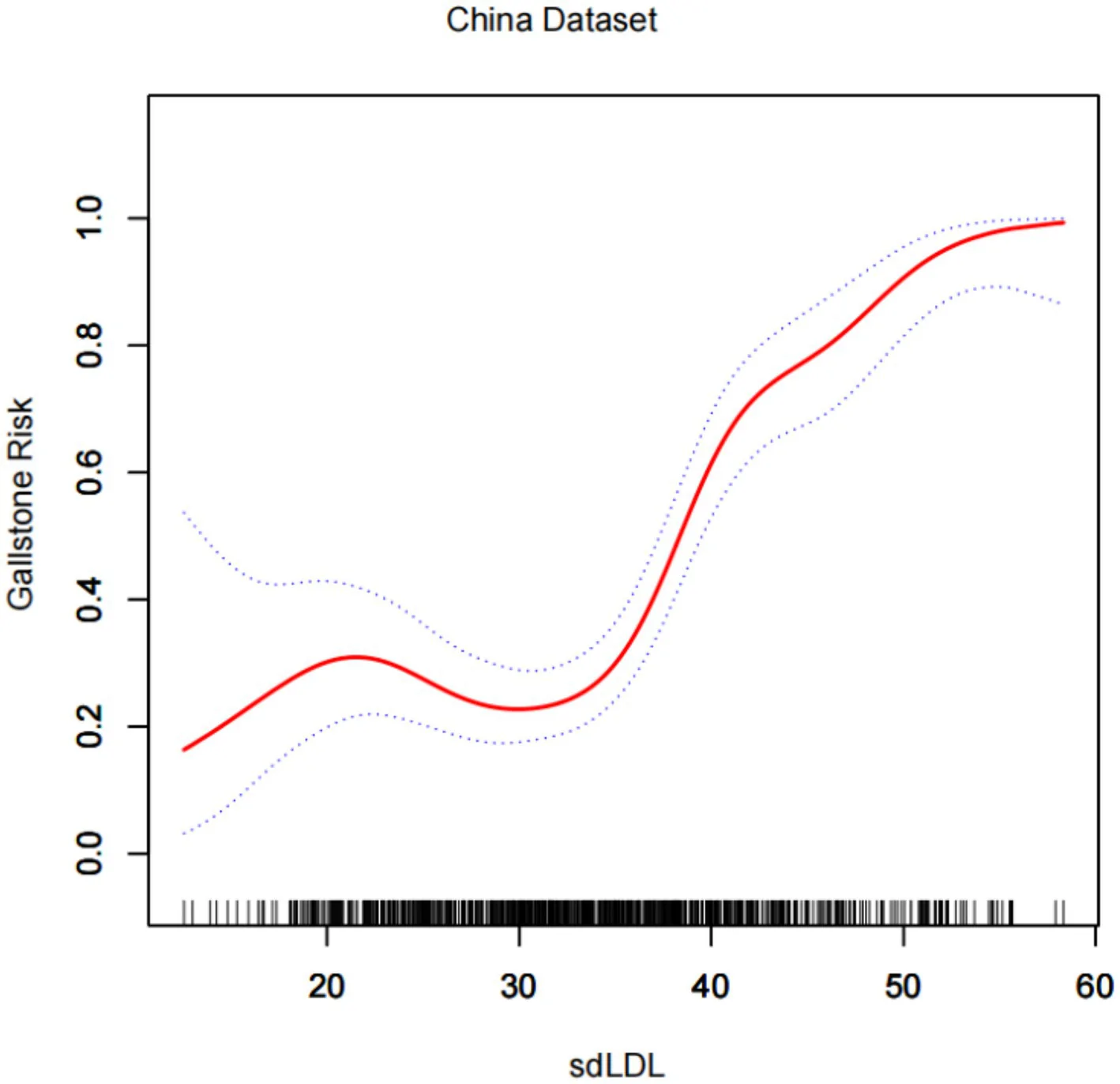
Dose-response relationship between sdLDL-C and gallstone risk in the Chinese single-center cohort.

**Figure 3:**
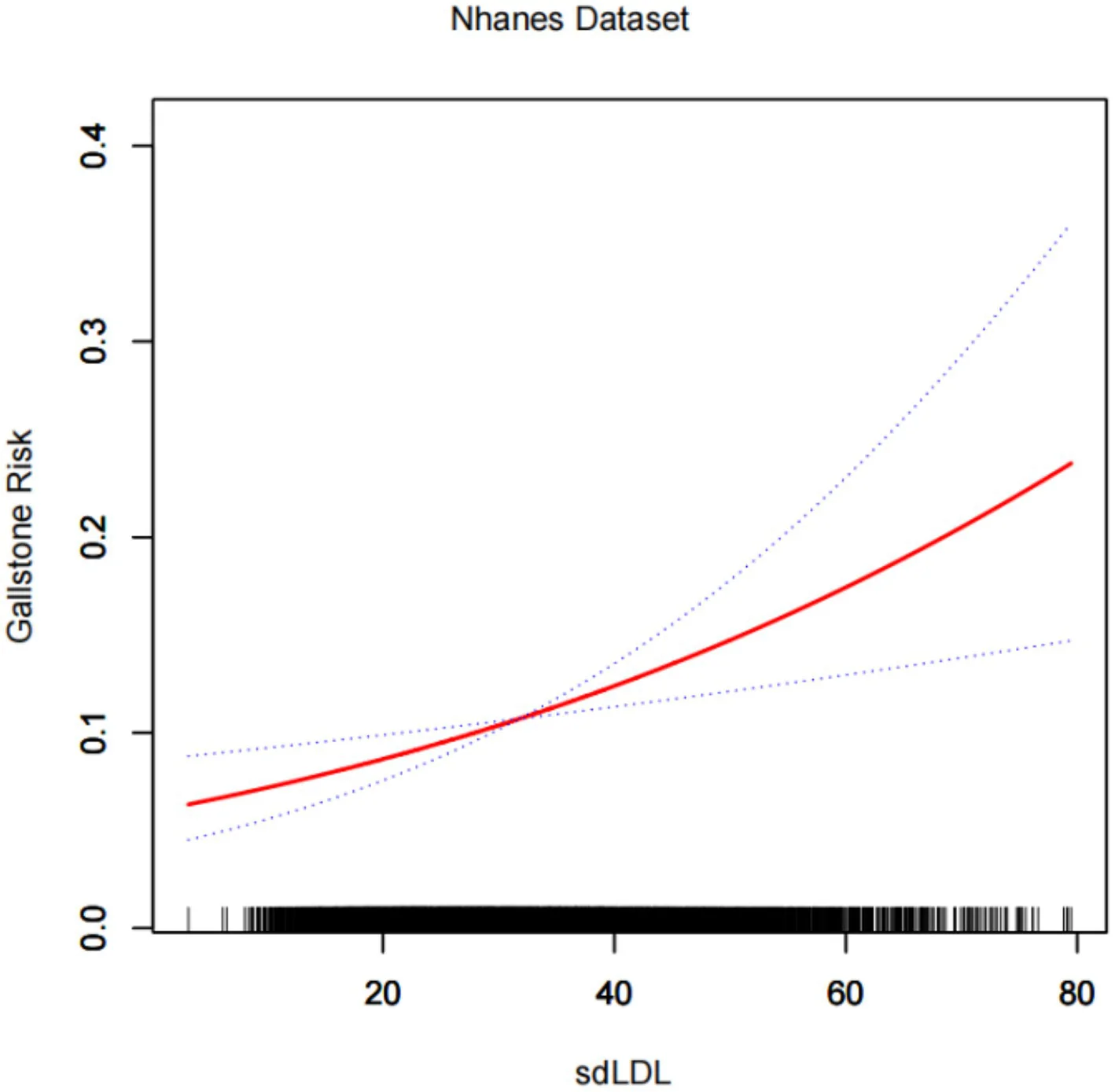
Dose-response relationship between sdLDL-C and gallstone risk in the NHANES cohort.

Smooth curve fitting and two-piecewise regression models were used to clarify the dose-response relationship (Table 4). Chinese Cohort (Non-linear Threshold Effect): Smooth curve fitting showed a clear curvilinear trend (Figure 2). The log-likelihood ratio test confirmed a significant non-linear relationship. Threshold effect analysis identified an inflection point at 34.51 mg/dL. For sdLDL-C < 34.51 mg/dL, the association with gallstone risk was not significant (OR = 0.99, 95% CI: 0.95–1.04, *p* = 0.812). For sdLDL-C ≥ 34.51 mg/dL, the risk increased sharply, with a 27% higher risk for each 1 mg/dL increase in sdLDL-C (OR = 1.27, 95% CI: 1.20–1.34, *p* < 0.001). NHANES Cohort (Linear Correlation): In contrast, the NHANES data demonstrated a steady linear positive correlation between sdLDL-C and gallstone risk, with no significant inflection point or threshold effect observed (Figure 3).

**Table 4:** Threshold effect analysis of sdLDL-C on gallstone risk in China data.

	Adjusted OR (95% CI)
Gallstone risk	
Total	1.12 (1.08, 1.15)
Fitting by two-piecewise logistic proportional risk model	
Inflection point	34.51
sdLDL-C < 34.51	0.99 (0.95, 1.04)
sdLDL-C ≥ 34.51	1.27 (1.20, 1.34)
*p* for Log-likelihood ratio	<0.001

### Subgroup analysis

Stratified analyses were performed to explore population heterogeneity and test for effect modifiers (Table 5). In the Chinese cohort, a significant interaction was observed for age (*p* for interaction = 0.02), indicating that the positive association between estimated sdLDL-C and gallstone risk was notably stronger in young adults (20–54 years: OR = 1.15, 95% CI: 1.11–1.20) compared to older adults. An interaction was also noted for hypertension status (*p* for interaction = 0.03). For diabetes, there was a marginally significant trend suggesting an amplified effect in diabetic patients (OR = 1.19, 95% CI: 1.08–1.31; *p* for interaction = 0.07). Notably, gender did not significantly modify the effect in the Chinese sample (*p* for interaction = 0.95). Conversely, in the NHANES cohort, the subgroup analysis revealed highly significant effect modification by gender (*p* for interaction = 0.001), with the positive association being distinctly prominent in females (OR = 1.03, 95% CI: 1.02–1.04) while remaining non-significant in males (OR = 1.01, 95% CI: 0.99–1.03). Similar to the Chinese cohort, the effect was also stronger in younger adults (20–54 years, *p* for interaction = 0.02).

**Table 5:** Stratified analysis of the association between sdLDL-C and gallstone risk.

Characteristic	Model 1 OR (95% CI)	Model 2 OR (95% CI)	Model 3 OR (95% CI)	*p* for interaction
China data				
Stratified by age (years)				0.02
20–54	1.11 (1.08, 1.13)	1.11 (1.08, 1.13)	1.15 (1.11, 1.20)	
55–85	1.07 (1.04, 1.10)	1.07 (1.04, 1.10)	1.06 (1.00, 1.11)	
Stratified by gender				0.95
Male	1.09 (1.06, 1.12)	1.09 (1.06, 1.12)	1.11 (1.06, 1.15)	
Female	1.09 (1.06, 1.12)	1.09 (1.06, 1.12)	1.14 (1.09, 1.20)	
Stratified by hypertension				0.03
No	1.08 (1.05, 1.10)	1.08 (1.05, 1.10)	1.12 (1.08, 1.16)	
Yes	1.13 (1.09, 1.17)	1.13 (1.09, 1.17)	1.09 (1.02, 1.16)	
Stratified by diabetes				0.07
No	1.08 (1.06, 1.11)	1.08 (1.06, 1.11)	1.11 (1.07, 1.14)	
Yes	1.14 (1.08, 1.21)	1.15 (1.08, 1.22)	1.19 (1.08, 1.31)	
NHANES data				
Stratified by age (years)				0.02
20–54	1.02 (1.01, 1.03)	1.02 (1.01, 1.03)	1.03 (1.01, 1.05)	
55–85	1.00 (0.99, 1.01)	1.00 (0.99, 1.01)	1.02 (1.01, 1.04)	
Stratified by gender				0.001
Male	0.99 (0.98, 1.00)	1.00 (0.98, 1.01)	1.01 (0.99, 1.03)	
Female	1.02 (1.01, 1.02)	1.01 (1.01, 1.02)	1.03 (1.02, 1.04)	
Stratified by hypertension				0.53
No	1.00 (0.99, 1.01)	1.00 (0.99, 1.01)	1.03 (1.01, 1.04)	
Yes	1.02 (1.01, 1.02)	1.01 (1.00, 1.02)	1.03 (1.01, 1.04)	
Stratified by diabetes				0.74
No	1.01 (1.00, 1.02)	1.01 (1.00, 1.02)	1.02 (0.99, 1.06)	
Yes	1.01 (1.00, 1.02)	1.01 (1.00, 1.01)	1.03 (1.01, 1.04)	

Model 1 = No covariates were adjusted.

Model 2 = Model 1 + age, gender.

Model 3 was adjusted for all covariates in Tables 1 or 2.

All statistical estimates (including means, frequencies, and odds ratios) for the NHANES cohort are survey-weighted to represent the US national population.

## Discussion

This study systematically observed an independent and significant positive association between elevated serum sdLDL-C levels and gallstone risk by integrating a Chinese single-center cohort and the US NHANES database. While previous epidemiological studies have noted associations between the overall lipid profile and gallstone disease (4).conventional lipid metrics (such as total LDL-C) often mask the true effect of atherogenic subfractions due to particle heterogeneity (8). The novelty of this study lies in highlighting sdLDL-C, a strong atherogenic marker, as a potential core factor associated with biliary metabolic imbalance. Importantly, in the sensitivity analysis (Model 4), this robust positive association persisted even after excluding patients with diagnosed hypertension, diabetes, and clinical dyslipidemia. This strongly suggests that estimated sdLDL-C may serve as an potential metabolic marker associated with gallstone risk, rather than merely a secondary manifestation of late-stage metabolic syndrome, and could represent a pathophysiological factor associated with cholesterol crystallization in the gallbladder.

A clinically translatable finding of this study is the marked heterogeneity in the dose-response relationship between the two studied datasets. In the Chinese cohort, a non-linear risk inflection point was identified at 34.51 mg/dL, beyond which the risk increased steeply. In contrast, based on the reliable US epidemiological data (9), the NHANES cohort showed a steady linear correlation. Rather than reflecting intrinsic biological or genetic variations between Eastern and Western populations, this difference in curve shapes is likely heavily influenced by the substantial methodological disparities between the two cohorts. Specifically, the Chinese dataset is a hospital-based case–control sample utilizing rigorous clinical imaging for symptomatic surgical patients, whereas NHANES is a general population cross-sectional survey relying on self-reported physician diagnoses. These fundamental differences in sampling strategies, outcome assessments, and baseline disease severities may largely account for the observed non-linear versus linear patterns. For instance, the unexpected finding of a significantly lower BMI among gallstone cases in the Chinese cohort is likely attributable to this specific clinical context: symptomatic patients awaiting surgery often experience recurrent biliary pain and voluntarily adopt strict dietary modifications (e.g., fat restriction), which can lead to preoperative weight loss compared to asymptomatic healthy controls. Furthermore, although this study estimated sdLDL-C using the Sampson equation, a highly accurate method validated in multiple large cohorts (6, 10), the 34.51 mg/dL inflection point observed in the Chinese sample should be interpreted strictly as an exploratory, cohort-specific observation rather than a definitive clinical threshold. Future validation in larger, prospective multicenter cohorts is mandatory before any universal clinical cut-offs for sdLDL-C can be established.

The underlying mechanisms that might explain the association between elevated sdLDL-C and cholesterol gallstones are likely multifactorial. First, hepatic lipid supersaturation and increased biliary excretion play a central role. High circulating sdLDL-C levels typically indicate hyperactive *de novo* lipogenesis in the liver and reduced peripheral clearance (11) Faced with this heavy atherogenic lipid burden, the liver actively transporting excess cholesterol into the biliary system. This shift in the lipid excretion pool directly leads to biliary cholesterol supersaturation and an abnormally elevated cholesterol saturation index (CSI), providing the primary physicochemical driving force for cholesterol monohydrate nucleation (12, 13). Thus, elevated peripheral sdLDL-C is a systemic reflection of local lipid supersaturation in the gallbladder lumen.

In addition to physical lipid accumulation, insulin resistance serves as a key amplifier in this lithogenic process. Subgroup analysis showed that the positive association of sdLDL-C was potentially amplified in diabetic patients. While these specific molecular pathways were not directly measured in our current epidemiological study, prior basic research has confirmed that hepatic insulin resistance not only fosters sdLDL particle generation but also relieves the suppression of the transcription factor FoxO1, thereby directly upregulating the expression of the cholesterol efflux transporters ABCG5 and ABCG8 on the apical membrane of cholangiocytes (14, 15). Concurrently, chronic hepatic insulin resistance significantly inhibits key enzymes in the bile acid synthesis pathway (e.g., CYP7B1) and impairs farnesoid X receptor (FXR) signaling, leading to a diminished pool of solubilizing bile acids (16). This “double hit” of massive cholesterol influx and severe bile acid depletion creates a highly lithogenic microenvironment, explaining the high gallstone risk in metabolically impaired populations (17).

Furthermore, the unique physicochemical properties of sdLDL particles may theoretically contribute to the deterioration of the biliary microenvironment. Being small and lacking antioxidants, sdLDL is highly susceptible to oxidative modification in circulation. These pro-inflammatory lipid metabolites easily infiltrate the microvasculature of the gallbladder wall, inducing chronic mucosal inflammation and severe oxidative stress (18). The cascade of inflammatory mediators stimulates epithelial cells to over-secrete mucin, providing a physical scaffold for crystal aggregation. It also directly impairs the contractile function of gallbladder smooth muscle cells, resulting in biliary stasis (19). Defective gallbladder emptying provides the essential anatomical “residence time” for potential microcrystals to grow into mature stones (20).

Regarding gender differences, the potentially stronger association observed in females suggests a possible synergistic exacerbating effect of sex hormones on the lipid-biliary metabolic axis. Physiologically, estrogen upregulates the activity of hepatic sterol excretion receptors. When this effect overlaps with the high lipid burden caused by sdLDL-C surges, it multiplies the lithogenicity of bile (21). However, it must be noted that the confidence intervals for males and females overlapped substantially in our subgroup analysis.

Despite the strengths of this study—including dual-cohort validation, a large sample size, and strict control of metabolic confounders through sensitivity analysis—several limitations should be acknowledged. First, the cross-sectional design restricts our ability to establish a definitive causal relationship between elevated sdLDL-C and gallstone formation. Reverse causality remains a potential issue; for instance, patients diagnosed with potential metabolic abnormalities may have adopted healthier diets or used statins, which could lower their current sdLDL-C levels and lead to an underestimation of its true lithogenic effect. The diagnosis of gallstones itself might induce dietary changes or altered enterohepatic circulation that affect lipid metabolism. Furthermore, patients diagnosed with potential metabolic abnormalities may have adopted healthier diets or unrecorded lifestyle interventions following their diagnosis. While current statin users were explicitly excluded from our analysis, the historical effects of past medications or lifestyle shifts could still exert residual influence on current lipid profiles, potentially leading to an underestimation of the true lithogenic effect. Future prospective longitudinal studies are needed to verify this causal chain.

Second, there is inherent heterogeneity in the diagnostic methods used across the two cohorts. The Chinese cohort utilized rigorous clinical imaging (ultrasound/CT), whereas NHANES relied on standardized self-reported questionnaires. While this diagnostic divergence might partially explain the differing shapes of the dose-response curves (threshold vs. linear) observed between the populations, it conversely serves as a powerful cross-validation of the primary hypothesis. However, the fact that the positive association of estimated sdLDL-C remained highly robust across these fundamentally different diagnostic modalities and racial backgrounds indirectly but strongly supports the generalizability and biological validity of our findings. Demonstrating consistent results across diverse clinical and epidemiological diagnostic standards suggests that the association is not an artifact of a specific study design, but rather a robust metabolic phenomenon.

Lastly, there are missing informational dimensions regarding precise variable measurement and pathological typing. We used the Sampson equation framework to estimate sdLDL-C and lbLDL-C; while highly accurate and cost-effective, it cannot completely replace direct physical measurement via ultracentrifugation. Since our Chinese clinical cohort was a retrospective analysis of electronic medical records, historical blood samples were unavailable for direct quantification. Nevertheless, applying the same validated equation uniformly across both the Chinese and US cohorts ensured methodological consistency and minimized measurement bias when comparing the cross-population dose-response curves. Additionally, due to the retrospective nature of the databases, data on gallstone chemical composition (e.g., pure cholesterol, black pigment, or mixed stones) were unavailable. Since lipid abnormalities primarily drive cholesterol stone formation, the lack of pathological typing limits our ability to analyze mechanisms in specific subgroups. The absence of detailed dietary fatty acid intake, specific bile acid pool compositions, and genetic polymorphisms of lipid transporters (such as ABCG5/8) also limits a comprehensive evaluation of the gallbladder microenvironment. Crucially, due to data limitations across both cohorts, our models did not adjust for several important potential confounders, including specific markers of visceral obesity (e.g., waist circumference), race/ethnicity, smoking status, alcohol consumption, menopausal status, physical activity, specific dietary patterns, socioeconomic status, gallbladder motility disorders, and the specific use of lipid-lowering medications or estrogen therapy. Finally, while this study mathematically derived lbLDL-C to estimate sdLDL-C according to the Sampson equation, we strictly focused our primary multivariable analytical models on the atherogenic sdLDL-C fraction to prevent severe statistical multicollinearity, as these mathematically coupled parameters would destabilize the regression estimates. Future prospective studies should simultaneously evaluate and contrast the lithogenic risks associated with standard LDL-C and large buoyant LDL-C (lbLDL-C) through direct physical quantification to fully map the specific lipid subfraction landscape in gallstone disease.

## Conclusion

In summary, estimated serum sdLDL-C is independently and positively associated with gallstone risk, exhibiting a significant non-linear risk threshold within a specific Chinese clinical cohort. Future multicenter prospective studies incorporating gallstone compositional analysis are warranted to elucidate the precise causal networks and molecular targets of sdLDL-C in biliary microenvironment deterioration, thereby providing better clinical guidelines for the precise metabolic risk stratification and clinical management of gallstone disease.

## Data Availability

The raw data supporting the conclusions of this article will be made available by the authors, without undue reservation.
